# Total pelvic exenterative surgery in patients with peritoneal metastases from appendiceal neoplasms. A case series of 2 patients

**DOI:** 10.1016/j.ijscr.2019.10.064

**Published:** 2019-11-05

**Authors:** Paul H. Sugarbaker

**Affiliations:** Program in Peritoneal Surface Malignancies, MedStar Washington Hospital Center, 106 Irving St., NW, Suite 3900, Washington, DC, 20010, USA

**Keywords:** Cytoreductive surgery, Hyperthermic intraperitoneal chemotherapy (HIPEC), Early postoperative intraperitoneal chemotherapy (EPIC), Pseudomyxoma peritonei, Low grade appendiceal mucinous neoplasms, High grade appendiceal mucinous neoplasms, Reoperative surgery

## Abstract

**Background:**

Surgery which includes peritonectomy, visceral resections, and perioperative chemotherapy has been used extensively to treat peritoneal metastases from appendiceal mucinous adenocarcinoma. The results of treatment vary with the grade of the neoplasm, the extent of abdominal and pelvic disease and the completeness of tumor removal.

**Methods:**

The clinical features, surgical procedures and outcome of two patients with mucinous appendiceal neoplasms were reviewed. The decision to move to total pelvic exenterative surgery after prior treatment failure was critically evaluated.

**Results:**

Patient 1 had three extensive surgical procedures prior to total pelvic exenteration and one surgical procedure after. Patient 2 had one 16-h surgical procedure prior to total pelvic exenteration. Patient 1 had 8 years and 1 month survival from onset of disease until death. Patient 2 is free of disease at 27 years from onset of pseudomyxoma peritonei. After recovery of the patients from the extensive surgery, their quality of life was acceptable.

**Conclusion:**

Total pelvic exenteration surgery is seldom used in patients with peritoneal metastases from appendiceal neoplasms. These two patients were studied in an attempt to determine the indications for this procedure with this disease.

## Highlights

• Pelvic exenteration is an unusual procedure in patients with peritoneal metastases from appendiceal cancer.
• Pelvic exenteration may be considered in a fit patient who is made visibly disease-free by surgery.
• More intensive local-regional chemotherapy may be considered in patients who fail cytoreductive surgery plus HIPEC and EPIC.
• Surgical features noted intraoperatively provide important information regarding a decision to proceed.

## 1 Introduction

Appendiceal mucinous neoplasms with peritoneal metastases are now treated with curative intent. The combination of complete cytoreductive surgery with perioperative intraperitoneal chemotherapy is currently the standard of care [1]. The most important requirement for long-term benefit is complete visible resection of all the disease [2]. The question often arises regarding the extent of the resections that is commensurate with the long-term benefit. In the primary disease setting a series of peritonectomy procedures is combined with visceral resections. Although these procedures may be extensive and require a long time in the operating room, the patient’s complete recovery with a normal quality of life is expected. In the reoperative setting long-term survival benefits are less frequent, but they do occur [3,4]. Also, impairment of quality of life may occur if additional visceral resections are required. Decisions regarding the risk of serious complications, and compromise of quality of life versus long-term survival with complete resection may be difficult.

In this case series two patients are subjected to total pelvic exenterative surgery as treatment for recurrent appendiceal mucinous neoplasms with peritoneal metastases. Clinical features associated with success are critically evaluated.

## 2 Materials and methods

The grade of the malignancy was assessed by an experienced pathologist as low, moderate or high grade [5]. The peritoneal cancer index (PCI) was scored at the time of the initial cytoreductive surgery as described by Jacquet and Sugarbaker [6]. The extent of peritoneal metastases was estimated as absent (0), less than 0.5 cm (1), 0.5–5.0 cm (2), and greater than 5.0 cm (3) in nine abdominopelvic regions and four small bowel regions. The score ranged from 0–39. The completeness of cytoreduction (CC) score was determined at the end of the cytoreductive surgery. It was scored for no visible tumor as CC-0, residual tumors less than 2.5 mm as CC-1, tumor nodules 2.5 mm–2.5 cm as CC-2, and residual nodules greater than 2.5 cm as CC-3 [6].

Data on these two patients was prospectively recorded and then retrospectively reviewed at an academic institution. This research work has been reported in line with the PROCESS criteria [7]. This study was registered as a case series on the www.researchregistry.com website with UIN 4834.

## 3 Patient presentation

### 3.1 Patient 1

In November 2008, this 46 year old woman noted a mass in the right lower quadrant of her abdomen. CT demonstrated a multicystic mass in the caecal/appendiceal region.

In December 2008, the patient underwent exploratory laparotomy. A large mass in the area of the appendix was visualized. Numerous mucinous tumor nodules were seen beneath right and left hemidiaphragm and within the greater omentum. A right hemicolectomy was performed. Pathology showed a high grade mucinous adenocarcinoma of the appendix with 3 of 17 lymph nodes positive for cancer.

In February 2009, a repeat CT scan showed an 8 cm right ovarian cyst and nodules within the greater omentum but was otherwise normal. A CEA was 10.1 ng/ml, CA19-9 was 50.7 μg/m, and CA125 was normal at 6.2 μg/ml. The patient returned to the operating room for cytoreductive surgery and perioperative chemotherapy. Procedures performed included greater and lesser omentectomy with splenectomy, right upper quadrant peritonectomy, and pelvic peritonectomy with hysterectomy [8]. The prior right ileocolic anastomosis required repeat resection. HIPEC with intraperitoneal mitomycin C and doxorubicin and systemic 5-fluorouracil was completed prior to closure of the abdomen [9]. Tube and drains were positioned for early postoperative intraperitoneal 5-fluorouracil. All specimens were positive for mucinous adenocarcinoma. The patient recovered without incident.

In July 2100, a CT scan showed 4 areas of disease progression. The CEA was 2.8 ng/ml and CA19-9 was 18.9 μg/ml. A third exploratory laparotomy was performed. Multiple thick fibrous adhesions were present. Three separate small bowel resections were required. A rectosigmoid colon resection was performed with low anastomosis. A solitary metastasis in the liver was resected. No perioperative chemotherapy was used. The procedure required 11 h. Metal clips were placed on a positive margin on the bladder. External beam radiation was used postoperatively in an attempt to control disease in the pelvis.

In February 2013, despite bladder irradiation, CT showed gross disease progression within the wall of the bladder. Right hydronephrosis had developed. The CEA was 4.3 ng/ml and CA19-9 was 42.5 μg/ml. A fourth exploratory procedure was performed. The exploration allowed visualization of the parietal peritoneum, liver, small bowel and large bowel. Disease progression was limited to the bladder with disease extension to the rectum and vagina. The bladder was excised and an ileal conduit was created. The old colorectal anastomosis was resected back to the junction of sigmoid and descending colon and an end sigmoid colostomy created. The procedure required 10 h.

In June 2015, the CEA had increased to 6.4 ng/ml and CA19-9 to 51.7 μg/ml. Enteric material was determined to be exiting from the vagina and CT showed a colovaginal fistula. The fistula was attributed to wide field pelvic radiation combined with extensive surgery. The colon in the pelvis which contained the fistula was excised and a more proximal colostomy created. Following recovery from this complication the patient gained weight and enjoyed a normal nutrition. Despite the colostomy and urostomy appliances, her activity level was satisfactory. She did not have a chronic pain syndrome.

The clinical and pathologic features for this patient are shown in Table 1. The treatment strategies used are presented in Table 2. The patient expired January 2016, eight years and 1 month after the diagnosis of high grade mucinous appendiceal cancer with peritoneal metastases and 3 years after her pelvic exenterative surgery.

**Table 1:** Clinical and pathologic features of two patients with appendiceal neoplasms having pelvic exenterative procedures. Data recorded at the time of initial cytoreductive surgery plus HIPEC. (HIPEC = hyperthermic intraperitoneal chemotherapy).

	Patient 1	Patient 2
Age	46	70
Gender	Female	Male
Primary site	Appendix	Appendix
Grade	High	Moderate
Lymph nodes	3/17 positive	None
Histologic type	Mucinous	Mucinous
Systemic metastases	None	None
Peritoneal cancer index	17	30
Completeness of cytoreduction	0	1
Neoadjuvant chemotherapy	None	None
CEA (ng/ml)	10.1	28.8
CA19-9 (units/ml)	50.7	143
Total survival	8 years, 1 month Dead from disease	27 years No evidence of disease

**Table 2:** Treatment strategies used in two patients with appendiceal neoplasms having pelvic exenterative procedures. (RUQ = right upper quadrant, LUQ = left upper quadrant, R = right, L = left, EPIC = early postoperative intraperitoneal chemotherapy, HIPEC = hyperthermic intraperitoneal chemotherapy).

	Patient 1	Patient 2
**Initial intervention:**		
Date	12/12/2008	02/18/1992
Indications	Appendiceal mass	Increased abdominal girth
Duration	3 h	16 h
Procedures	R hemicolectomy Partial omentectomy	Peritonectomy RUQ, LUQ, and pelvis, R and L colectomy, partial gastrectomy, greater and lesser omentectomy, splenectomy
Perioperative chemotherapy	None	EPIC
Blood replacement packed RBCs	None	8
Adverse events (Grade 3 and 4)	None	None

**Second intervention:**		
Date	02/25/2009	09/05/2008
Indications	R ovarian cyst, omental nodules	CT showed abdominal and pelvic masses
Duration	11 h	13 h
Procedures	Omentectomy, peritonectomy RUQ and pelvis, splenectomy, hysterectomy and oophorectomy, liver wedge resection	Peritonectomy, cystectomy, ileostomy and ileal conduit
Perioperative chemotherapy	HIPEC and EPIC	HIPEC
Blood replacement packed RBCs	None	5
Adverse events (Grade 3 and 4)	None	None

**Third intervention:**		
Date	07/13/2011	
Indications	Rising CEA, nodules by CT	
Duration	11 h	
Procedures	3 separate small bowel resections, rectosigmoid colon resection, postoperative bladder irradiation	
Perioperative chemotherapy	None	
Blood replacement packed RBCs	None	
Adverse events (Grade 3 and 4)	None	

**Fourth intervention:**		
Date	02/06/2013	
Indications	Cystoscopy-confirmed recurrence in bladder	
Duration	10 h	
Procedures	Small bowel resection, cystectomy, ileal conduit	
Perioperative chemotherapy	None	
Blood replacement packed RBCs	None	
Adverse events (Grade 3 and 4)	None	

**Fifth intervention:**		
Date	06/17/2015	
Indications	Colovaginal fistula	
Duration	4 h	
Procedures	Colon resection	
Perioperative chemotherapy	None	
Blood replacement packed RBCs	None	
Adverse events (Grade 3 and 4)	None	

### 3.2 Patient 2

In January 1992, a 53 year old man noted increasing abdominal girth and constipation for several months. The CEA was 28.8 ng/ml and CA19-9 was 143 μg/ml. The CA125 was 30.8 μg/ml. CT showed ascites and omental thickening. Paracentesis produced mucoid fluid and cells consistent with mucinous carcinoma. Colonoscopy was normal and barium enema showed a filling defect in the appendix.

In February 1992, with a diagnosis of pseudomyxoma peritonei the patient had a cytoreductive surgery. The procedure required 16 h. There was a greater and lesser omentectomy with splenectomy, peritoneal stripping of right and left hemidiaphragm and pelvis, right colon and rectosigmoid colon resections with anastomosis and distal gastrectomy with gastrojejunostomy. Tubes and drains were placed for early postoperative intraperitoneal chemotherapy (EPIC) [8]. All specimens showed moderate grade mucinous adenocarcinoma. The tumor was invasive.

The patient recovered well. He received 6 cycles of combined intraperitoneal 5-fluorouracil and systemic mitomycin C [9].

In September 2008, CT showed approximately 8 tumor nodules within the small bowel regions and a confluence of tumor in the pelvis. The CEA was 6.6 ng/ml and CA19-9 was 39.3 μg/ml. The CA125 was 4.4 μg/ml. The patient required resection of tumor nodules from the posterior aspect of the stomach, resection of numerous tumor nodules from the small bowel, total proctocolectomy with end ileostomy and total cystectomy with an ileal conduit. Heated intraoperative intraperitoneal chemotherapy (HIPEC) was used.

The clinical and pathologic features for this patient are shown in Table 1. The treatment strategies used are presented in Table 2.

Hospital stay was 18 days. Rehabilitation was complete. The patient has no evidence of disease as of February 2019. He is now 27 years from his initial diagnosis and disease-free with an excellent quality of life. He maintains his urostomy and ileostomy on his own. His nutritional status remains normal. He does not have a pain syndrome and takes no pain medicine on a regular basis.

## 4 Discussion

The decision to proceed with resection of both bladder and rectum (total pelvic exenteration) was not simple in either of these patients. In patient 1, the only palliative option for bladder recurrence with ureteral obstruction seem to be resection. This decision was reinforced by a negative exploration of the abdomen. It is possible, in retrospect that ureteral stents would have provided palliation. This exceptionally radical surgery did not result in a long-term disease-free survival. Also, a serious complication, probably as a result of extensive surgery that was performed on irradiated tissues, occurred 28 months later. This was a colovaginal fistula.

The second patient recurred with a long free interval of 16 years. At exploration several sites of disease were documented outside of the pelvis. These were removed and treated with HIPEC with no recurrence [9]. The decision to remove the entire colon with bladder resulted in long-term benefit. In patient 2, the reoperative surgery plus HIPEC was effective in the control of microscopic residual disease.

In patient 1, the HIPEC plus EPIC did not control microscopic residual disease at the time of the first cytoreduction. Perioperative chemotherapy was not used in subsequent cytoreductive procedures. Perhaps a greater effort with perioperative chemotherapy should have occurred in an attempt to preserve the visible complete repeat cytoreduction.

Appendiceal mucinous neoplasms may metastasize extensively to parietal peritoneal surfaces and the greater omentum. Origin of a malignancy in the right lower quadrant (appendix) is an unusual anatomic site for an extensive resection within the pelvis. Usually, the indication for this surgery is a pelvic malignancy such as cervical cancer [10], rectal cancer [11], or bladder cancer [12]. No prior reports of total pelvic exenteration for appendiceal adenocarcinoma peritoneal metastases has been published.

The peritoneal metastases do accumulate and then progress within the pelvis in large volume with mucinous appendiceal neoplasms. With a large extent of cancer resected by the original cytoreduction and despite HIPEC plus EPIC, recurrence in the pelvis was seen. In both patients the pelvis was visibly free of tumor at the completion of the first cytoreductive surgery. The progression of gross disease at this site indicates that the mucinous tumor was not sufficiently responsive to the hyperthermic intraperitoneal chemotherapy. Alternatively, the distribution of HIPEC and EPIC was such that insufficient contact of chemotherapy solution and residual microscopic disease within the pelvis occurred. A major shortcoming of the intraperitoneal chemotherapy in both of these patients is the single application of the chemotherapy treatment. Recent attempts to repeat the intraperitoneal chemotherapy treatments by drug instillation in a large volume of fluid directly into the peritoneal space have been published [13,14]. Plans to use repeat chemotherapy instillations in gastrointestinal cancer with peritoneal metastases are in progress.

Perhaps the most important observations from this study regards the clinical, radiologic, pathologic and surgical features of these patients that led to total pelvic exenteration surgery. These two patients were both a performance status one and thought to be fit for a major surgery plus perioperative chemotherapy. Radiologic study by CT with both oral and intravenous contrast showed an absence of concerning radiologic features [15]. There was one exception to this in patient 1. She had obstruction of the right ureter; ureteral obstruction is one of the 15 concerning radiologic features that are radiologic contraindications to complete cytoreduction. In this patient the ureteral obstruction was relieved by cystectomy and construction of an ileal conduit.

The surgical exploration must be complete if total pelvic exenteration is being considered. Unresectable disease in the abdomen would be a contraindication to such an extensive resection. To perform total pelvic exenteration and have residual disease at another site would defy established surgical principles. In patient 1, complete exploration revealed no disease outside of the pelvis. In patient 2, there were tumors resected from small bowel surfaces and from the posterior aspect of the stomach. Tumor on the bladder, colon and rectum was definitively resected by total proctocolectomy plus total colectomy.

Finally, the mucinous adenocarcinoma, as expected, did not metastasize out of the abdominal space to distant lymph nodes, to liver or to systemic sites. The positive lymph nodes present in the right colon specimen in patient 1 was a warning that the disease may have an invasive capability difficult, perhaps impossible to eradicate by cytoreductive surgery and single cycle of perioperative chemotherapy with hyperthermic mitomycin C intraoperatively and normothermic intraperitoneal 5-fluorouracil immediately postoperatively. Multiple cycles of intraperitoneal chemotherapy may have been beneficial in both of these patients.

## Funding

Secretarial support and data management support funded by Foundation for Applied Research in Gastrointestinal Oncology.

## Ethical approval

Local IRB-approval for this case report was not required:

MedStar Health Institutional Review Board has determined that a case report of less than three (3) patients **does not meet the DHHS definition of research** (45 CFR 46.102(d)(pre-2018)/45 CFR 46.102(l)(1/19/2017)) **or the FDA definition of clinical investigation** (21 CFR 46.102(c)) and therefore are not subject to IRB review requirements and **do not require IRB approval**.

This case report is only of 2 patients.

## Consent

Written and signed consents were obtained from the patients.

## Author’s contribution

Paul H. Sugarbaker, MD: study concept or design, data collection, data analysis or interpretation, writing the paper.

## Registration of research studies

This case report is registered as a case series on the www.researchregistry.com website with UIN 4834.

## Guarantor

Paul H. Sugarbaker, MD.

## Provenance and peer review

Not commissioned, externally peer-reviewed.

## Declaration of Competing Interest

The author has no conflicts of interest to declare.
